# Society Meetings

**Published:** 1911-07

**Authors:** 


### SOCIETY MEETINGS

The Ontario Medical Association at its thirtieth annual meeting held at Niagara Falls, Ont., June 1, 1911, elected the following named officers for the ensuing year: President Herbert A. Bruce, Toronto; vice presidents, F. W. E. Wilson, Niagara Falls; William Hall, Brampton; F. P. Drake, London, and George Field, Cobourg; treasurer, J. Hourner Mullin, Hamilton; secretary, F. Arnold Clarkson, Toronto.

The Lackawanna Railroad Surgeons' Association held its annual meeting in Buffalo in June. Thirty-two members were present representing all the larger towns between New York and Buffalo. The meeting was presided over by Dr. Richard T. Bang, of New York, president of the association. The care of railroad injuries was the subject most generally discussed. First aid material and several new appliances for emergency cases were shown. By invitation, Dr. E. R. McGuire, of Buffalo, read a paper before the association. The members were entertained at luncheon by Dr. F. J. Carr, the Buffalo member.

The American Medical Editors' Association held its forty-second annual meeting at Los Angeles, June 26 and 27, under the presidency of Dr. J. MacDonald, Jr., of New York. Among the papers presented were the following: Relation of the Medical Press to the Public Health and Marine Hospital Service, by Walter Wyman, Surgeon General, U. S. Public Health and Marine Hospital Service; The Advisibility of Newspapers and Magazines having Medical Editors on Their Staff, by Edgar A. Vander Veer; The Literary Side of Medical Journalism, by T. D. Crothers; Private Owned Medical Journals, by Henry W. Coe.

The American Dermatological Association at its thirty-fifth annual meeting held at the Hotel Lenox, Boston, May 25, 26 and 27, 1911, elected the following-named officers for the ensuing year: president, Grover W. Wende, of Buffalo; vice president, James S. Howe, of Boston; secretary and treasurer, James M. Winfield, of Brooklyn; councillor-at-large, William A. Pusey, of Chicago. St. Louis was selected as the next place of meeting.

